# Variability in the response of canine and human dendritic cells stimulated with *Brucella canis*

**DOI:** 10.1186/s13567-017-0476-8

**Published:** 2017-11-02

**Authors:** Myriam Pujol, Francisca Castillo, Carla Alvarez, Camila Rojas, Consuelo Borie, Arturo Ferreira, Rolando Vernal

**Affiliations:** 1 Doctoral Program in Agronomy Forestry and Veterinary Sciences, Faculty of Veterinary Sciences, Universidad de Chile, Santiago, Chile; 2 Periodontal Biology Laboratory, Faculty of Dentistry, Universidad de Chile, Santiago, Chile; 3 Laboratory of Veterinary Bacteriology, Department of Animal Preventive Medicine, Faculty of Veterinary Sciences, Universidad de Chile, Santiago, Chile; 4 Program of Immunology, Institute of Biomedical Sciences ICBM, Faculty of Medicine, Universidad de Chile, Santiago, Chile; 5 Dentistry Unit, Faculty of Health Sciences, Universidad Autónoma de Chile, Santiago, Chile

## Abstract

*Brucella canis* is a small intracellular Gram-negative bacterium whose primary host is the dog, but it also can cause mild human brucellosis. One of the main causes of an inefficient immune response against other species of *Brucella* is their interaction with dendritic cells (DCs), which affects antigen presentation and impairs the development of an effective Th1 immune response. This study analysed the cytokine pattern production, by RT-qPCR and ELISA, in human and canine DCs against whole *B. canis* or its purified LPS. Human and canine DCs produced different patterns of cytokines after stimulation with *B. canis*. In particular, while human DCs produced a Th1-pattern of cytokines (IL-1β, IL-12, and TNF-α), canine cells produced both Th1 and Th17-related cytokines (IL-6, IL-12, IL-17, and IFN-γ). Thus, differences in susceptibility and pathogenicity between these two hosts could be explained, at least partly, by the distinct cytokine patterns observed in this study, where we propose that human DCs induce an effective Th1 immune response to control the infection, while canine DCs lead to a less effective immune response, with the activation of Th17-related response ineffective to control the *B. canis* infection.

**Electronic supplementary material:**

The online version of this article (10.1186/s13567-017-0476-8) contains supplementary material, which is available to authorized users.

## Introduction

*Brucella canis*, a small Gram-negative facultative intracellular coccobacillus, belonging to the *Brucella* genus and *Proteobacteria* phylum, is responsible for canine brucellosis [1, 2]. It is a worldwide distributed zoonotic pathogen whose prevalence is unknown, because routine human brucellosis diagnosis does not include *B. canis* detection [3].

Canine brucellosis is considered the main cause of reproductive failures in dogs [4], provoking late abortions in females and epididymitis and/or prostatitis in male dogs, leading to infertility, and non-reproductive symptoms such as ophthalmitis, diskospondylitis, lymphadenopathy, and splenomegaly [1, 2, 5]. Both, infected dogs and healthy dogs harbouring *B. canis* have the potential to transmit the bacteria to humans, mainly by oronasal contact, occupational interaction with infected animals, or laboratory accidents [6–9]. When humans are infected, *B. canis* frequently induce mild or asymptomatic infections that may remain undiagnosed for protracted periods [3, 10–12] and clinical signs include headache, recurrent fever, weakness, fatigue, chills, sweats, and weight loss [13].

There is strong evidence indicating the relevance of dendritic cells (DCs) function in the pattern of inflammatory-immune response triggered against other *Brucella* species [14–18]. Due to the differences in the clinical manifestation between dogs and human during *B. canis* infection, this study aims to assess whether their DCs, when exposed to *B. canis,* produce different patterns of immune response. We hypothesized that *B. canis* interacts differently with human and canine DCs leading to distinct pattern of cytokine expression, thus contributing to explain, at least partially, the different susceptibility and pathogenicity observed in dogs and humans.

## Materials and methods

### *Brucella canis* isolation and characterization

The *B. canis* strain SCL was isolated from an urine sample of a male stray dog which had been recently admitted to a dog shelter in Santiago, Chile. The dog had clinical history of urinary symptoms, including inflammation, pain and haematuria, and was positive to a counterimmunoelectrophoresis serological test using *B. ovis* LPS antigen [19]. Bacterial growth was performed in *Brucella* agar 5% sheep blood and morphological and biochemical characteristics were compatible with *B. canis*, including CO_2_ requirement, oxidase, urease, catalase, citrate utilization, sugar fermentation, and H_2_S production [20]. The *B. canis* strain SCL was fully sequenced (Next-Generation Illumina MiSeq Platform^®^; Illumina Inc., San Diego, CA, USA), at the Favet-Inbiogen Laboratory belonging to the Faculty of Veterinary Sciences, Universidad de Chile, under the GenBank accession number LGAQ00000000.1. Finally, the *Brucella* genus was confirmed by qPCR, using a Real Time PCR Detection Kit (Genesig^®^; Southampton, UK) in a StepOnePlus^®^ qPCR equipment (Applied Biosystems, Singapore).

### *Brucella canis* growth conditions and curves

The *B. canis* strain SCL was cultured on 5% bovine serum tripticase soy agar or 5% horse blood agar (Oxoid Ltd, Hampshire, UK) and incubated at 37 °C under aerobic conditions. Bacterial growth curves were obtained in 5% bovine serum tripticase soy broth (Oxoid Ltd) incubated at 37 °C with constant orbital shaking. Briefly, bacterial samples were inoculated in 4 mL of broth until reaching an optical density (OD) of 0.05, as measured by spectrophotometry at a wavelength of 560 nm (Halo RB-10; Dynamica Scientific Ltd., United Kingdom). The spectrophotometry readings were taken every 1 h and the experiment was stopped when *B. canis* reached the stationary growth phase (30 h). With each OD measurement, a sample was taken, log_10_ serially diluted in PBS, and 100 µL of each dilution were cultured on tripticase soy agar and incubated at 37 °C. Finally, the number of colony-forming units/mL (CFU/mL) counted was plotted against the corresponding OD reading. For DC stimulation with whole *B. canis*, bacteria were taken at the exponential growth phase in order to obtain a reliable number of live bacteria having their whole antigenic potentiality.

### *Brucella canis* LPS purification

For DC stimulation with purified lipopolysaccharide (LPS), *B. canis* LPS was obtained using a modified version of the TRIzol reagent protocol as described previously [21]. Briefly, *B. canis* was cultured in 200 mL of tripticase soy broth until it reached the stationary growth phase and was immediately pelleted by centrifugation at 6000* g* for 10 min. The pellet was washed with distilled water and then incubated in 3 mL of TRIzol reagent (TRIzol^®^ Plus; Invitrogen Corp., Barcelona, Spain) and 600 µL chloroform (Fluka, Sigma-Aldrich Chemie GmbH, Buchs, Switzerland) at room temperature for 30 min. After centrifugation, the aqueous phase was treated with proteinase K, RNase and DNase (Sigma-Aldrich, St. Louis, MO, USA) and lyophilized overnight. The obtained LPS was then resuspended in 1 mL of 95% ethanol with 0.375 M MgCl_2_, washed three times with cold 100% ethanol, and purified with 1% Folch reagent containing 2:1 chloroform and methanol. Finally, the LPS was dried overnight in a fume cabinet, quantified using the malondialdehyde thiobarbituric acid reaction, resuspended in milli-Q water as stock solution, and stored at −20 °C [21]. LPS was visualized by sodium dodecyl sulphate-14% polyacrylamide gel electrophoresis (SDS-PAGE) and periodic acid-silver staining as described previously [22]. Coomassie blue stained gels were used to determine absence of protein contamination. *B. canis* manipulation was carried out inside a biosafety cabinet and approved by the Biosafety Committee from North Campus, Universidad de Chile.

### Peripheral blood samples

Peripheral blood samples were obtained from 10 healthy human and 10 healthy dogs. For human samples, buffy coats were obtained from healthy female and male consenting adults from the Clinical Hospital José Joaquín Aguirre, Universidad de Chile. An extensive anamnesis was performed and criteria for individual selection were as follows: absence of fever or manifest infections during the last month, absence of concomitant systemic disease or pregnancy, and no medical history of brucellosis. Further exclusion criteria were positive tests for human immunodeficiency virus, hepatitis B or C viruses, symptomatic allergies, abnormal blood cell counts, increased liver enzymes, or medication of any kind except vitamins and oral contraceptives. The study was approved by the Ethics Committee on Human Research, Universidad de Chile. Dog blood samples were obtained by venipuncture into heparin tubes (Vacutainer^®^; Becton–Dickinson and Company, New Jersey, NJ, USA) after clinical, hematological and biochemical evaluation, together with counterimmunoelectrophoresis serological analysis, using *B. ovis* LPS antigen [19], in order to discard the presence of brucellosis. Dog donors were female and male belonging to private owners, aged 1–7 years, regularly dewormed and vaccinated against distemper, leptospirosis, parvovirus, parainfluenza, canine adenovirus type 2, canine hepatitis, and rabies. Dog owners provided written informed consent approved by the Committee for Animal Care and Use, Universidad de Chile, and all procedures were conducted according to Institutional Ethical Guidelines.

### Dendritic cell generation

Human and canine DCs were generated using a three-step protocol as described previously [23]. First, peripheral blood mononuclear cells (PBMCs) were isolated by density gradient centrifugation using standard procedures (Ficoll-Paque Plus^®^; GE Healthcare, Uppsala, Sweden). Second, monocytes were purified from PBMCs by magnetic cell sorting using an anti-CD14 monoclonal antibody conjugated to magnetic beads (MACS^®^; Miltenyi Biotec, Bergisch Gladbach, Germany). Third, DCs were generated by culturing CD14^+^ monocytes at 1 × 10^6^ cells/mL for 6 days in RPMI-1640 (Life Technologies, Gaithersburg, MD, USA), supplemented with 10% foetal bovine serum, 100 U/mL penicillin, 100 µg/mL streptomycin, 2 mM l-glutamine (Gibco Invitrogen Corp., Grand Island, NY, USA), 100 U/mL polymixin B (Sigma-Aldrich, St. Louis, MO, USA), and specific recombinant human (rh) or canine (rc) differentiation factors. In particular, canine monocyte cultures were supplemented with 40 ng/mL rhGM-CSF and 30 ng/mL rcIL-4 (R&D Systems Inc., Minneapolis, MN, USA), while human monocyte cultures were supplemented with 20 ng/mL rhGM-CSF and rhIL-4 (R&D Systems Inc.) every 2 days.

### Dendritic cell stimulation

Differentiated DCs were then primed with live whole *B. canis* (MOIs = 2 × 10^−1^ to 2 × 10^2^) or *B. canis* purified LPS (10, 100 ng/mL or 1 µg/mL) for 24 h. Dendritic cells stimulated with 1 µg/mL LPS of *Escherichia coli* strain 0128:B12 (Sigma-Aldrich, St. Louis, MO, USA) and non-stimulated DCs were used as controls. For each subject, the experiments were performed separately. In each experimental step, cell counting was performed in a Neubauer^®^ chamber using a phase contrast microscopy (AxioVert 100; Carl Zeiss Co., Göttingen, Germany) and cell viability was calculated by Trypan blue dye exclusion.

### Phenotypic analysis of dendritic cell generation and activation by flow cytometry

The canine and human monocyte differentiation towards DCs and their activation in the presence of *B. canis* were analyzed by flow cytometry as described previously [23]. Differentiated DCs were stained with anti-CD1a, CD11c, and CD14 monoclonal antibodies (BD Biosciences Pharmingen, San José, CA, USA) for 30 min at 4 °C in the dark and then analyzed using a flow cytometer (BD LSR Fortessa™; Becton–Dickinson, Pleasanton, CA, USA). In order to compare the activation and maturation levels of human DCs upon stimulation with *B. canis*, the expression levels of CD83 (specific marker of DC maturation) and CD86 (costimulatory signal expressed by DCs and necessary for T-lymphocyte activation during antigen presentation) were determined. In turn, the activation and maturation levels of canine DCs were determined by analysis of the expression of CD86 and DLAII (dog leucocyte antigen class II). Data from 6 independent experiments are expressed as mean ± SD, percentage of CD-positive cells and mean fluorescence intensity (MFI) for each surface marker.

### Morphologic cell analysis of stimulated dendritic cells by scanning electron microscopy

The morphological changes observed in *B. canis*-stimulated human or canine DCs were analyzed by scanning electron microscopy. Briefly, DCs were fixed with 2% glutaraldehyde for 2 h, washed three-times with PBS, dehydrated through immersion in graded ethanol (50, 70, 95 and 100%), and dried at CO_2_ critical point. Cells were then mounted on aluminium stubs, sputter-coated with gold layer to a thickness of 200 nm (Denton Vacuum Desk V; Moorestown, NJ, USA), and examined in a scanning electron microscope (Jeol JSMIT300LV; Jeol Ltd, Tokyo, Japan) at an accelerating voltage of 20 kV.

### Expression of cytokines by RT-qPCR

After whole bacteria or LPS stimulation for 24 h, total cytoplasmic RNA was isolated from DCs using 400 µL of ice-cold lysis buffer containing 0.5% Igepal^®^ CA-630 (Sigma-Aldrich, Saint Louis, MO, USA), 50 mM Tris–HCl pH8, 100 mM NaCl, 5 mM MgCl_2_, and 10 mM VRC-40 (Gibco Invitrogen, Carlsbad, CA, USA) as described previously [23]. Isolated RNA was quantified using a spectrophotometer (Synergy HT; Bio-Tek Instrument Inc., Winooski, VT, USA) and the first-strand cDNA strand was synthesized using 5 µg of total RNA and a reverse transcription kit following the manufacturer’s protocol (Maxima First Strand cDNA Synthesis kit for RT-qPCR^®^; Thermo Scientific, Waltham, MA, USA). The mRNA expression for the cytokines IL-1β, IL-4, IL-5, IL-6, IL-10, IL-12p35, IL-13, IL-17, IL-23, IFN-γ, TNF-α, and TGF-β1 were analyzed by RT-qPCR. Briefly, 50 ng of cDNA were amplified using the appropriate canine (Additional file 1) or human primers (Additional file 2) and the KAPA™ SYBR^®^ Fast qPCR reagent (KAPA Biosystems, Woburn, MA, USA) in a StepOnePlus^®^ qPCR equipment (Applied Biosystems) as follows: 95 °C for 3 min, followed by 40 cycles of 95 °C for 3 s and 60 °C for 30 s, and finally a melting curve of 95 °C for 15 s, 60 °C for 1 min and 95 °C for 15 s, for detection of non-specific product formation and false-positive amplification. Calibration curves were performed and a primer matrix was carried out to optimize the concentration of each primer, also in order to confirm the gene specificity for a single amplification product, a Blast search of the selected primers was performed in the Gene Bank sequence database. In human DCs, 18S ribosomal RNA (18S rRNA) expression levels were used as endogenous control. Due to insufficient background regarding the adequate choice of reference gene for cytokine expression in canine DCs, 3 frequently recommended reference genes were analyzed: 18S rRNA, glyceraldehyde 3-phosphate dehydrogenase (GAPDH), and TATA box binding protein (TBP) [24–26]. The expression level stability for these reference genes was analyzed using the NormFinder algorithm [27]. Data from 10 independent experiments are shown as mean ± SD. Each experiment was performed in duplicate.

### Secretion of cytokines by ELISA

In addition, DC culture supernatants were collected and ELISA analysis was carried out to determine the secretion of human IL-12p70 and TNF-α (Quantikine^®^; R&D Systems Inc.) and canine IL-17 and IFN-γ (Nori Canine^®^; Genorise Scientific Inc., Philadelphia, PA, USA). Plates were quantified in an automatic microplate spectrophotometer (Synergy HT; Bio-Tek Instrument Inc.) according the manufacturer’s instructions. Data from 7 independent experiments are shown as mean ± SD.

### Statistical analysis

The flow cytometry data were analyzed using the FlowJo software v9.6.4 (TreeStar, Ashland, ORE, USA), represented as histograms, and expressed as the percentage of positive cells over the total. The RT-qPCR data were analyzed using the StepOne Software v2.2.2 (Applied Biosystems) and the relative quantification was obtained by normalizing the cytokine expression to endogenous control expression using the 2^−ΔΔCt^ method. The ELISA data were calculated using a four-parameter logistic equation. Data were expressed as mean ± SD and statistically analyzed using the GraphPad Prism software v5.0 (GraphPad software Inc., La Jolla, CA, USA). The normality of data distribution was determined using the Kolmogorov–Smirnov test. The differences between groups were determined using the one-way ANOVA test followed by Tukey or Bonferroni post hoc test. Statistical significance was assumed when *p* < 0.05.

## Results

### *B. canis* characterization and growth curves

**Figure 1:**
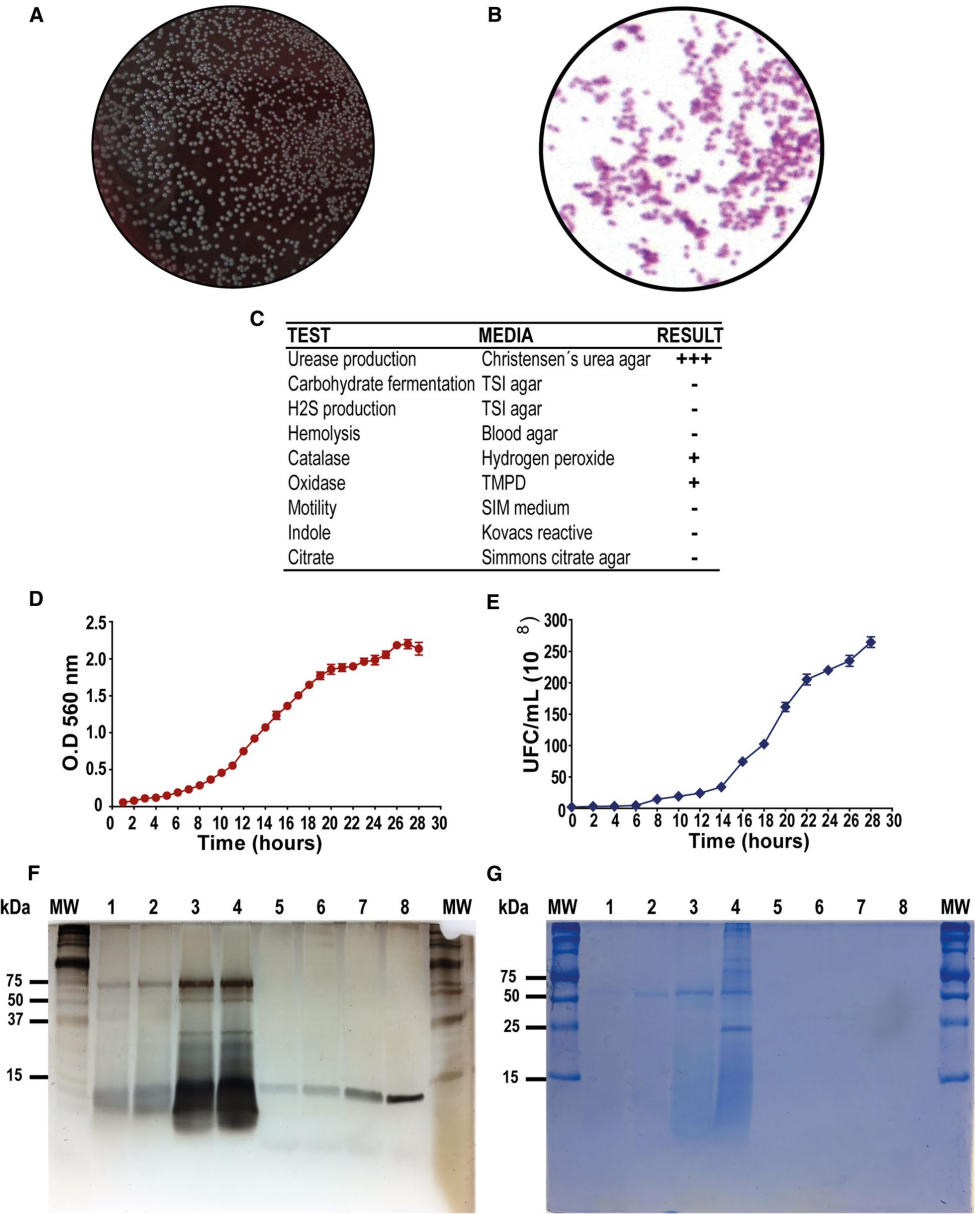
***Brucella canis*** **microbiological characterization, LPS isolation and growth curves.** Colonies growing on 5% horse blood agar (**A**), bacteria observed under optical microscopy (**B**), and biochemical profile analyzed under different conditions (**C**). Bacterial growth curves plotted as time versus optical density (OD) (**D**) and time versus colony-forming units/mL (CFU/mL) (**E**). Silver stained SDS-PAGE (**F**) and Comassie blue gels (**G**) of whole cellular membrane lysate of *B. canis* (lanes 1–4) and its purified LPS (lanes 5–8).

The microbiological characterization of *B. canis* strain SCL used in the present study is shown in Figure 1. In culture, *B. canis* grew in convex, circular colonies of 1–2-mm diameter (Figure 1A), and under optical microscopy, bacteria behaved as small Gram-negative coccobacillus (Figure 1B). The biochemical profile was consistent with that described for other clinical isolates of *B. canis* [28, 29]; in particular, bacteria were catalase and oxidase positive, and showed urease production before 30 s post-induction (Figure 1C). Bacterial growth curves plotted as OD versus time and CFU/mL versus time are shown in Figures 1D and E, respectively.

### *Brucella canis* LPS is compatible with rough LPS

When the whole cellular membrane lysates from *B. canis* were analyzed (lanes 1–4), silver stained SDS-PAGE (Figure 1F) revealed some upper bands that could be confused with the presence of O-polysaccharide; however, the detection of this bands in Coomassie blue stained gels (Figure 1G) strongly suggest that they correspond to protein structures. When the LPS purified from *B. canis* was analyzed (lanes 5–8), only bands compatibles with LPS from rough bacteria (core and lipid-A) were detected, and the upper bands were not present in both the silver stained SDS-PAGE gel and the Coomassie blue gel. In general terms, the use of the TRIzol based protocol resulted effective for obtaining a purified LPS from rough *B. canis.*

### Highly pure human and canine dendritic cell cultures were obtained

**Figure 2:**
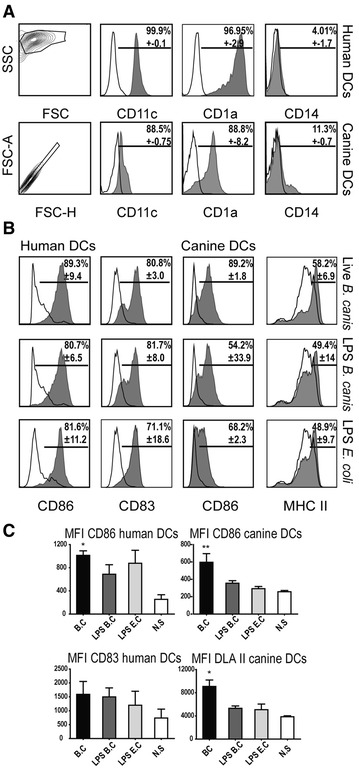
**DC cultures purity and activation.** DC differentiation from blood purified monocytes in the presence of GM-CSF and IL-4 was identified by flow cytometry according to forward scatter (FSC) and side scatter (SSC) parameters. Gated human and canine DCs were analyzed for expression of CD1a, CD11c, and CD14 and *B. canis*-stimulated labelled DCs (shaded histograms) were compared to FMO (fluorescence minus one) control (black solid line) (**A**). Activation and maturation levels of DCs after stimulation with *B. canis*: human and canine DCs were identified according to FSC and SSC parameters, and gated cells were analyzed for expression of CD86, CD83, and DLA II (shaded histograms) and compared to non-stimulated cells (black solid line) (**B**). Mean fluorescence intensity (MFI) for each surface marker shown in **B** (**C**). *p < 0.05 and  **p < 0.01.

The DC differentiation was demonstrated by the combined staining with anti-CD1a, CD11c, and CD14 monoclonal antibodies analyzed by flow cytometry (Figure 2A). Dendritic cells were identified by FSC and SSC parameters, and debris or doublets were excluded from the analyses. About 95% of the obtained human cell cultures were positive for the superficial markers CD1a and CD11c, while around 90% of the canine cells resulted positive for these two markers. In addition, canine and human DCs expressed very low and un-detected levels of the monocyte-macrophage marker CD14, respectively, thus indicating that highly purified populations of differentiated DCs were used for *B. canis* stimulation.

### Stimulation with *B. canis* induces activation and maturation of canine and human dendritic cells

**Figure 3:**
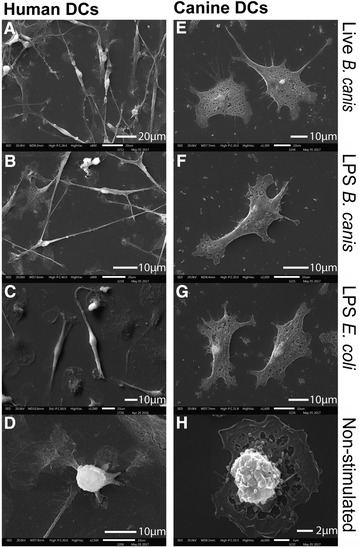
**Morphologic changes in DCs after stimulation with** ***B. canis*** **or its purified LPS.** Scanning electron microscopy analysis of human or canine DCs stimulated for 24 h with live *B. canis* or it purified LPS. Human *B. canis*-stimulated DCs (**A**) developed numerous long projections and their cell size was at least five times greater than non-stimulated DCs (**D**). LPS-stimulated DCs (**B**, **C**) also increased cell size and developed projection in a less intense manner. Canine stimulated DCs (**E**–**G**) also showed increased cell size and developed projections. Non-stimulated human (**D**) and canine (**H**) DCs were round shaped with short projections.

Both human and canine DCs showed upregulation of maturation and activation surface markers upon stimulation with *B. canis*. When human or canine DCs were stimulated with whole *B. canis*, a high proportion of cells increased the expression levels of CD86 (98.6 and > 90.9%, respectively) and human CD83 (77.8%) or canine MHC II (58.2%) (Figure 2B). A less intensive increment in all markers was detected when DCs were stimulated with *B. canis* purified LPS, without differences as compared to *E. coli* LPS (Figure 2C). Scanning electron microscopy analysis showed morphological changes compatible with signs of cell activation in stimulated human and canine DCs, including increased cell size and development of long cell projections (Figure 3).

### The 18S rRNA translates a suitable housekeeping gene and allows quantification of cytokine expression by RT-qPCR in canine dendritic cells

Due to insufficient background regarding the adequate choice of reference genes for quantification of cytokine expression in canine DCs, the expression levels of three potential housekeeping genes were measured for RT-qPCR normalization (Table 1). Primer pairs were designed to identify the 18S rRNA, GADPH, and TBP expression in 28 canine samples (Additional file 1) and the NormFinder algorithm was used for identifying the optimal normalization gene among the three selected candidates, according to stability of expression [27]. The algorithm analysis revealed that 18S rRNA was the most stably expressed housekeeping gene, with a stability value of 0.304. Therefore, 18S rRNA was used as reference gene for RT-qPCR analyses.

**Table 1:** **Housepeeking genes for quantification of cytokine mRNA expression using RT-qPCR in canine DCs**

Target	Stability value	Standard error
GAPDH	0.659	0.186
TBP	1.475	0.210
18S rRNA	0.304	0.350

*GAPDH* glyceraldehyde 3-phosphate dehydrogenase, *TBP* TATA box binding protein, *18S rRNA* subunit 18S ribosomal RNA.

### Canine dendritic cells elicit a Th1/Th17-pattern of cytokine expression while human cells elicit a Th1-pattern of cytokines upon *B. canis* stimulation

**Figure 4:**
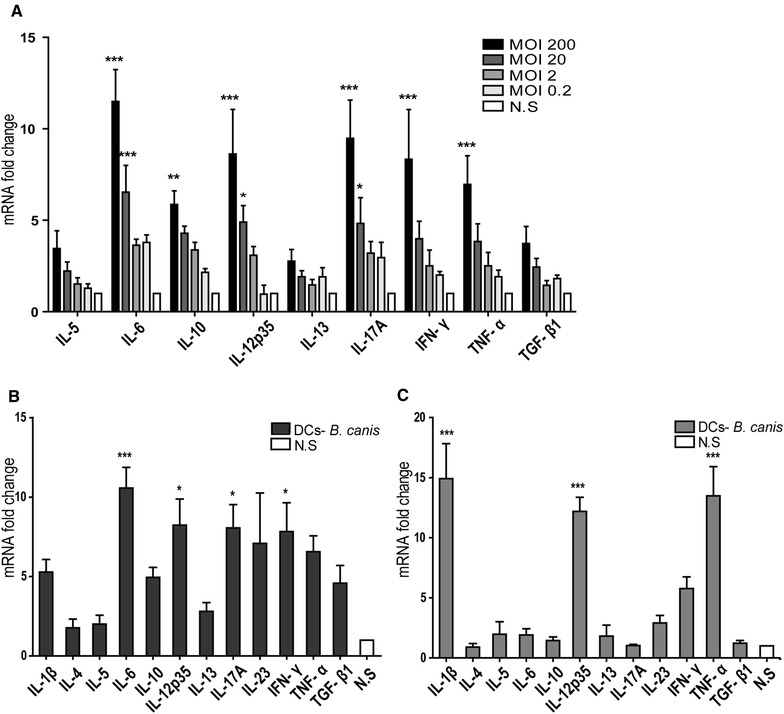
**Different cytokine mRNA expression in whole** ***B. canis*** **-stimulated canine or human DCs.** Quantification of the cytokines IL-1β, IL-12p35, IFN-γ, and TNF-α (Th1-type), IL-4, IL-5, and IL-13 (Th2-type), IL-6, IL-17, and IL-23 (Th17-type), and IL-10 and TGF-β1 (T regulatory-type) is represented as mRNA fold-change in DCs stimulated with whole *B. canis*. Dose-dependent increase in the cytokine mRNA expression when canine DCs were stimulated at MOIs = 2 × 10^−1^ to 2 × 10^2^ (**A**). Cytokine mRNA expression in canine (**B**) and human DCs (**C**), when cells were stimulated with whole *B. canis* at a MOI = 2 × 10^2^. The cytokine mRNA expression in non-stimulated DCs was considered as 1, as a reference for fold-change in expression (N.S.). *p < 0.05, **p < 0.01, and ***p < 0.001.

Figure 4 depicts the expression of pro-inflammatory cytokines in DCs stimulated with whole *B. canis*. The data, plotted as mRNA fold-change for each cytokine, showed a dose-dependent increase in the mRNA expression levels for IL-5, IL-6, IL-10, IL-12p35, IL-13, IL-17, IFN-γ, TNF-α, and TGF-β1 (Figure 4A). When canine DCs were exposed to *B. canis*, an increment in the mRNA expression for the Th1-associated cytokines (IL-12 and IFN-γ) and the Th17-associated cytokines (IL-6 and IL-17) was detected as compared to non-stimulated DCs (Figure 4B). Conversely, only the Th1-associated cytokines (IL-1β, IL-12, and TNF-α) were over-expressed in infected human DCs as compared to non-stimulated cells (Figure 4C).

### *B. canis* LPS stimulation induced a Th1/Th17-pattern of cytokine expression in canine DCs and a Th1-pattern of cytokines in human DCs

**Figure 5:**
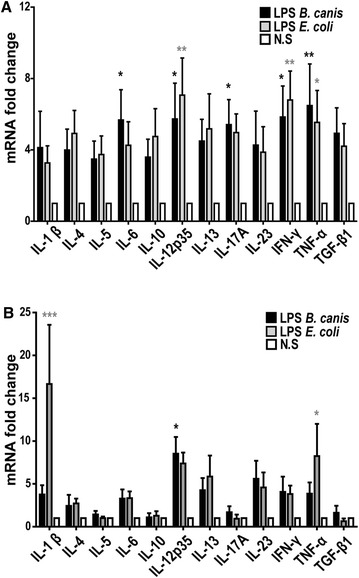
**Cytokine mRNA expression in** ***B. canis*** **LPS-stimulated DCs.** Quantification of the cytokines IL-1β, IL-12p35, IFN-γ, and TNF-α (Th1-type), IL-4, IL-5, and IL-13 (Th2-type), IL-6, IL-17, and IL-23 (Th17-type), and IL-10 and TGF-β1 (T regulatory-type) is represented as mRNA fold-change in canine (**A**) and human (**B**) DCs stimulated with 1 µg/mL of *B. canis* purified LPS. DCs stimulated with 1 µg/mL LPS of *E. coli* strain 0128:B12 were used as controls. The cytokine mRNA expression in non-stimulated DCs was considered as 1, as a reference for fold-change in expression (N.S.). *p < 0.05 and **p < 0.01.

Figure 5 depicts the expression of pro-inflammatory cytokines in DCs stimulated with *B. canis* purified LPS (1 µg/mL). An increment in mRNA expression for the Th1-associated cytokines (IL-12, IFN-γ, and TNF-α) and the Th17-associated cytokines (IL-6 and IL-17) was detected in canine DCs stimulated with *B. canis* LPS as compared to non-stimulated DCs, and these increased cytokine levels were similar than those detected in *E. coli* LPS-stimulated DCs (Figure 5A). Conversely, only the Th1-associated cytokine IL-12 was over-expressed in LPS *B. canis*-stimulated human DCs as compared to non-stimulated cells (Figure 5B). Lower doses of *B. canis* purified LPS were also used (10 and 100 ng/mL) with slight or no effects on canine DC activation.

### When stimulated with *B. canis,* canine and human dendritic cells elicit Th1/Th17 and Th1-pattern of cytokines, respectively

**Figure 6:**
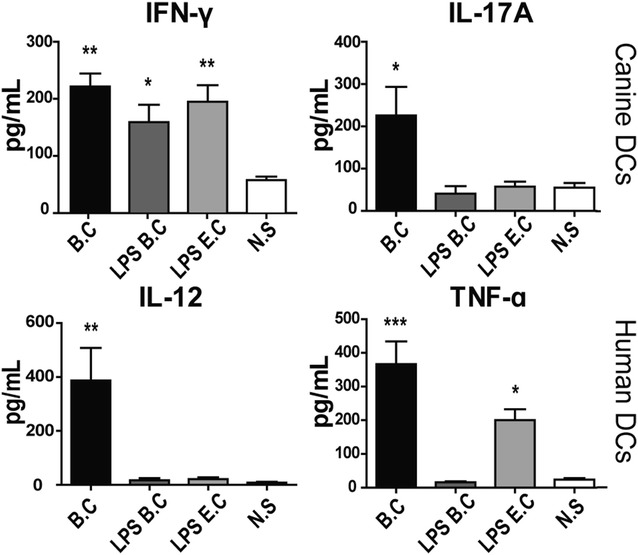
**Cytokine secretion in DCs stimulated with** ***B. canis***, ***B. canis*** **LPS or** ***E. coli*** **LPS.** Quantification of human IL-12 and TNF-α (Th1-type) (**A**) and canine IFN-γ and IL-17 (Th1 and Th17-type) (**B**) is represented as pg/mL in DCs stimulated with live *B. canis* or LPS. Non-stimulated DCs were used as control (N.S.). *p < 0.05, **p < 0.01, and ***p < 0.001.

The described changes in the cytokine mRNA levels were consistent with the changes in protein levels evaluated by ELISA (Figure 6). A significant increment in the secreted levels of Th1 or Th17-associated cytokines (IFN-γ and IL-17) was confirmed in canine DCs stimulated with whole *B. canis*, while stimulation with *B. canis* or *E. coli* purified LPS induced increased secretion of IFN-γ, as compared to non-stimulated DCs. An increment of IL-12 was detected in *B. canis*-stimulated human DCs and also TNF-α production was detected in *B. canis* and *E. coli* LPS-stimulated DCs.

## Discussion

Brucellosis, a disease that affects different animal species, is caused by bacteria of the genus *Brucella* and some of them have zoonotic potential such as *B. melitensis*, *B. abortus*, *B. suis*, and *B. canis* [30]. Although these bacteria have the ability to infect humans, *B. canis*, less infective than the others, is associated with fewer symptomatic diseases and low number of reported cases [1–3]. It has been demonstrated that a large number of *B. canis* interacts with canine placental tissues and immune cells from different hosts [31–36]; however, there is no evidence of interaction between *B. canis* and human or canine DCs. Several studies have reported that *B. canis* has the ability to infect human and murine phagocytes, but failed to demonstrate intracellular proliferation [31, 33, 35]; however, when bacteria are inoculated at the exponential growth phase, they can proliferate in murine macrophages [37].

In this study, peripheral monocytes from healthy donors were purified and then differentiated into DCs to analyze the effects of whole *B. canis* or its purified LPS and to compare the production of pro-inflammatory cytokines between canine and human cells. The differentiation and activation of DCs was associated with the loss of the monocyte-macrophage marker CD14 and increase in CD1a, CD11c, CD83, DLAII, and CD86 expression, a pattern consistent with previous studies [23, 38, 39]. In addition, similar levels of DC activation were reached when stimulated with either whole bacteria, demonstrating that *B. canis* induces canine and human DC activation to a similar extent (Figure 2). Morphological changes consistent with signs of cell activation were also detected by scanning electron microscopy analyses (Figure 3). Thus, the differential DC response triggered shown between dogs and humans may be explained by qualitative rather than quantitative differences in the DC activation, and these differences may be caused by the differential immunogenicity demonstrated by *B. canis*. The present data indicate that *B. canis* induced an immune response biased towards both a Th1 and Th17 pattern of cytokine production in canine DCs; however, an immune response biased only towards a Th1 pattern of cytokine production was detected in human DCs (Figures 4, 6). The same cytokine patterns were detected when canine or human DCs were stimulated with *B. canis* purified LPS, demonstrating that its LPS contributes at least in part to the immune response detected against *B. canis* (Figures 5, 6).

Dendritic cells are key components of the innate immune response and act as a bridge for the adaptive immune response [40]. They are antigen-presenting cells that circulate through the bloodstream in immature states and are scattered in nearly all tissues. Immature DCs capture microbial antigens and then turn into mature cells with the ability to stimulate naïve T-lymphocytes, determining their Th-polarization and activation pattern, what in turn may define the disease phenotype, in particular whether a mild or severe infection is established [41]. The differential host-dependent pathogenic potential of *B. canis* observed between dogs and humans could be explained, at least in part, by the differences in the pattern of cytokine production in stimulated DCs detected in the present study.

It has been reported that an effective immune response against *Brucella* spp. is associated with the Th1-pattern cytokine production by induced DCs and T lymphocytes [15, 18, 42]. In fact, DCs are key target cells for *Brucella* spp., playing a central role in both the innate recognition and the establishment of a robust adaptive response. Different *Brucella* spp., however, invade these cells, impairing their capability to eliminate intracellular bacteria and the development of an adequate Th1 immune response [43].

Although in the present study it was detected an increment in the Th1 promoting cytokines IL-12 and IFN-γ in *B. canis*-stimulated canine DCs, it was also detected a significant increment in the Th17 polarizing cytokines IL-6 and IL-17, which was an interesting finding due to the lack of previous reports demonstrating the production of IL-17 by canine DCs. In this context, *B. canis*-stimulated human DCs over-expressed exclusively Th1 polarizing cytokines IL-1β, IL-12, and TNF-α (Figures 4, 6), suggesting that this pattern could lead to an effective Th1 immune response. Production of IL-12 and TNF-α by macrophages and DCs has been directly related with the control *B. abortus* infection [44, 45] and IL-1β promotes macrophage activation impairing replication of intracellular bacteria such as *Mycobacterium tuberculosis* by a process mediated by TNF-α secretion and TNFR1 cell surface expression [46]. Interestingly, Th17-type cytokines have been widely described in phagocytes stimulated with different species of *Brucella* [31, 47, 48]; however, the interpretation of these type of immune response in canine DCs can be complicated, considering that on one hand Th17 is not a fully differentiated phenotype and remain some cell plasticity, which can lead to a protective or harmful immune response in different cases [49]. On the other hand, although the Th17 subset in dogs has been described and associated with chronic inflammation [50], there is not enough background in this field of canine immunology.

Th17 lymphocytes are considered a potent pro-inflammatory and osteoclastogenic cell population, closely associated with autoimmune disorders [48]. In addition, although the Th17-related immune response can be effective against extracellular bacterial or fungal infections, it is considered ineffective to control intracellular bacterial infections [51, 52]. The Th17 cell subset has been associated with the development of osteoarticular lesions in different hosts infected by *Brucella* spp. [31, 48, 53], although it has been also suggested that the combination Th1/Th17 response induced by oral vaccines would be suitable to control *Brucella* spp. infection [47, 54]. There is evidence indicating that only a Th1 response is protective against *B. melitensis* and the development of a Th17 combined immune response do not affect the course of infection [55]. One possible explanation for these opposite effects is that the Th17 protective role refers to a local immune response in the gut, where Th17 lymphocytes usually play an important defensive role maintaining the mucosal homeostasis and integrity [52, 56]. In fact, there is evidence that an oral vaccine can lead to a protective Th1/Th17 response that protects mice from an oral administration of live *Brucella*, but the parenteral administration of the same antigen lead to a Th1 response, that protects mice from a systemic challenge [54].

*Mycobacterium tuberculosis* is a widely studied intracellular bacterium that induces a protective Th1 response, with the parallel development of a detrimental Th17 response that contributes with the clinical symptoms and favours the formation of granulomas in affected organs [57]. It is well known that IL-17 promotes granulopoiesis [52], although some *Brucella* spp. are differently resistant to neutrophil response [58]; furthermore, different *Brucella* spp., including *B. canis*, are able to resist killing by bovine neutrophils [59]. Considering that the main consequences of *B. canis* infection are osteoarticular lesions such as diskospondylitis and the presence of granulomatous lesions in affected organs [1, 60, 61], it is possible to assume that the presence of IL-6 and IL-17 could lead to a Th17-pattern of immune response, contributing to the brucellosis pathogenicity in infected dogs. In fact, increased serological levels of IL-17 are detected in human patients affected of acute brucellosis and these levels decrease after receiving antibiotic treatment, which indicates this cytokine is directly involved with the pathogenesis of human brucellosis [62].

In human Th lymphocytes, the Th17 cell plasticity can lead to different subsets, including IL-17/IL-10 secreting cells that in some cases can be protective for some bacterial infections; however, Th17 cells can also acquire the ability of IL-17/IFN-γ production that promote chronicity in inflammatory disorders [56, 63]. When Th17 knockout mice were infected with *B. melitensis*, no effect was described after elimination of this cell subset, revealing that Th17 cells may not affect the course of the infection [55]. In relation to this, although Th17 lymphocytes have been implicated with pathogenicity of rheumatoid arthritis, many IL-17 targeted therapies have failed to control the disease signs and symptoms [64]. However, the use of antibodies that neutralize TNF-α, a cytokine that can bias Th17 cells towards pathogenic Th1/Th17 cells, can specifically decrease this cell subset, improving symptomatic lesions [56, 63]. This indicate that a variety of Th17 cells can be induced and differentially affect the immune response.

The results of the present investigation strongly suggest that the differences in the cytokine patterns observed between canine and human DCs could have an impact in the development of the *B. canis* infection. Thus, a more effective Th1 immune response is displayed in human, while a Th1/Th17 immune response is displayed in dogs, impairing the elimination of the bacteria and favoring its pathogenicity. The involvement of the Th17-type immune response could also explain the long periods of asymptomatic relapses, because these cells are characterized by long periods of dormant phases, followed by short periods of cell activation. Taken together, these data contribute at least in part to elucidate the canine brucellosis immunopathology and to a better understanding of canine immunology through the description of IL-17 producing canine DCs.

## Additional files

**Additional file 1.** **Canine primers.** Canine forward and reverse primers for cytokine and endogenous control amplifications by RT-qPCR.

**Additional file 2.** **Human primers.** Human forward and reverse primers for cytokine and endogenous control amplifications by RT-qPCR.
