# Supplementary material for: Variability in the response of canine and human dendritic cells stimulated with Brucella canis
Source: Vet Res. 2017 Nov 2;48:72. doi: 10.1186/s13567-017-0476-8 (PMC5667440; doi:10.1186/s13567-017-0476-8)
Supplement: Supplementary file 1 — Additional file 1. Canine primers. Canine forward and reverse primers for cytokine and endogenous control amplifications by RT-qPCR. [file 13567_2017_476_MOESM1_ESM.docx]

**Additional file 1** **Canine forward and reverse primers for cytokine and endogenous control amplifications by RT-qPCR.**

| Target | Forward Primer | Reverse Primer | Accession Number |
| --- | --- | --- | --- |
| IL-1β | tacctgtggtcttgggcatc | tctagctgtagggtgggctt | NM_001037971.1 |
| IL-4 | gcttactagcactcaccagca | tcgtttctcgctgtgaggatg | NM_001003159.1 |
| IL-5 | acctgcaagtatttcttggtgtaa | aagccggtttgttctcaactt | NM_001006950.1 |
| IL-6 | tggctactgctttccctacc | ttgaagtggcatcatccttg | NM_001003301.1 |
| IL-10 | gcaccctacttgaggacgac | agctctcggagcatgtgg | NM_001003077.1 |
| IL-12p35 | cagagcaacagatggagcaa | ttattaactccattcaaaagcaactg | NM_001003305.1 |
| IL-13 | tgatcaatgtctccgactgc | acagtgctttcagcatcctct | NM_001003384.1 |
| IL-17A | gctccccagagcagacttt | aagaaccctaatgagtttagtcagaaa | NM_001165878.1 |
| IL-23 | gactcacagaacggacagca | tcaaatctggctggctctgg | XM_538231.5 |
| IFN-γ | gttgctgcctacttgggaac | ggcgtctgacatgcctcta | NM_001003174.1 |
| TNF-α | tcacttcctctgacccctca | agccctgagcccttaattct | NM_001003244.4 |
| TGF-β1 | tacattgacttccgcaagga | gttagcgtggtaacccttgg | NM_001003309.1 |
| GAPDH | gatgggcgtgaaccatgaga | tggtcatggatgactttggct | NM_001003142.2 |
| TBP | taagagagccccgaaccact | ttcttcactcttggctcccg | XM_014118233.1 |
| 18S rRNA | ctcaacacgggaaacctcac | cgctccaccaactaagaacg | 2ZKQ_A |

GAPDH: glyceraldehyde 3-phosphate dehydrogenase; TBP: TATA box binding protein; 18S rRNA: subunit 18S ribosomal RNA.
