# Supplementary material for: Variability in the response of canine and human dendritic cells stimulated with Brucella canis
Source: Vet Res. 2017 Nov 2;48:72. doi: 10.1186/s13567-017-0476-8 (PMC5667440; doi:10.1186/s13567-017-0476-8)
Supplement: Supplementary file 2 — Additional file 2. Human primers. Human forward and reverse primers for cytokine and endogenous control amplifications by RT-qPCR. [file 13567_2017_476_MOESM2_ESM.docx]

**Additional file 2** **Human forward and reverse primers for cytokine and endogenous control amplifications by RT-qPCR.**

| Target | Forward Primer | Reverse Primer | Accession Number |
| --- | --- | --- | --- |
| IL-1β | ctgtcctgcgtgttgaaaga | ttgggtaatttttgggatctaca | NM_000576.2 |
| IL-4 | caccgagttgaccgtaacag | gccctgcagaaggtttcc | NM_000589.3 |
| IL-5 | ctctgaggattcctgttcctgt | cagtacccccttgcacagtt | NM_000879.2 |
| IL-6 | gcccagctatgaactccttct | gaaggcagcaggcaacac | NM_000600.4 |
| IL-10 | tgggggagaacctgaagac | ccttgctcttgttttcacagg | NM_000572.2 |
| IL-12p35 | cactcccaaaacctgctgag | tctcttcagaagtgcaagggta | NM_000882.3 |
| IL-13 | agccctcagggagctcat | ctccataccatgctgccatt | NM_002188.2 |
| IL-17A | tgggaagacctcattggtgt | ggatttcgtgggattgtgat | NM_002190.2 |
| IL-23 | agcttcatgcctccctactg | ctgctgagtctcccagtggt | NM_016584.2 |
| IFN-γ | ggcattttgaagaattggaaag | tttggatgctctggtcatctt | NM_000619.2 |
| TNF-α | cagcctcttctccttcctgat | gccagagggctgattagaga | NM_000594.3 |
| TGF-β1 | cacgtggagctgtaccagaa | cagccggttgctgaggta | NM_000660.6 |
| 18S rRNA | ctcaacacgggaaacctcac | cgctccaccaactaagaacg | NR_145820.1 |

18S rRNA: subunit 18S ribosomal RNA.

# 
